# Elevated Hsp90-beta contributes to differential diagnosis of pleural effusion caused by lung cancer and correlates with malignant biological behavior of lung cancer

**DOI:** 10.1186/s12890-018-0752-z

**Published:** 2018-12-06

**Authors:** Rong Biaoxue, Li Min, Fu Tian, Gao Wenlong, Liu Hua

**Affiliations:** 1 Department of Respiratory Medicine, First Affiliated Hospital, Xi’an Medical University, 48 Fenghao West Road, Xi’an, 710077 China; 2 Department of Respiratory Medicine, Shenmu Hospital, Shenmu, China; 3 Department of respiratory Medicine, Jining NO.1 People’s Hospital, Jining, China; 4 Institute of Epidemiology and Health Statistics, School of Public Health, Lanzhou University, Lanzhou, China; 5 Department of Respiratory Medicine, Gansu Provincial Hospital, Lanzhou, China

**Keywords:** Lung cancer, Malignant pleural effusion, MPE, Hsp90-beta, Biomarker, Diagnosis

## Abstract

**Background:**

Hsp90-beta has been investigated to be correlated with the occurrence and development of tumor. The intention of this research was to test the level of Hsp90-beta in malignant pleural effusion (MPE) of patients with lung cancer and disclose the clinical significance of Hsp90-beta as a potential tumor marker for differential diagnosis of pleural effusion caused by lung cancer.

**Methods:**

The level of Hsp90-beta was determined using enzyme-linked immunosorbent assay. Calculations of the Hsp90-beta threshold, the sensitivity and specificity for distinguishing MPE from benign pleural effusion were performed using receiver operator characteristic curve.

**Results:**

The level of Hsp90-beta in MPE of lung cancer patients was higher than that in control individuals (*P* < 0.05) and increased MPE Hsp90-beta was correlated with the pathological differentiation, tumor size and lymphatic metastasis (*P* < 0.05). The cutoff value of Hsp90-beta produced by receiver operator characteristic curve for distinguishing lung cancer from control individuals were 1.659 ng/mL and the sensitivity and specificity were 93.46 and 79%.

**Conclusions:**

Increased Hsp90-beta in MPE was correlated with malignant biological behavior of lung cancer patients, indicating that the level of Hsp90-beta could be a tool of referential value for differential diagnosis of pleural effusion caused by lung cancer.

## Introduction

With the development of molecular biology, the research results at the molecular level have made great influence on molecular diagnosis and molecular targeted therapy of tumors, and new drugs for different targets can be designed at the molecular level [1]. It is believed that early confirmation of lung cancer will reduce mortality and improve cure rate. Therefore, finding the valuable diagnostic biomarkers are very important to improve the clinical outcome of lung cancer [2]. Malignant pleural effusion (MPE) is a common complication of lung cancer. The devastating effects of MPE, such as dyspnea and cough, severely impair the quality of life (QOL) of patients, and indicate a shorter survival [3]. The use of pleural effusion in the diagnosis of lung cancer has attracted the attention of clinical medical workers, for example, thoracic hydrocytology is very important for the diagnosis of MPE, but its sensitivity is limited (30–60%). However, studies on the genome, transcriptology, methylation and proteomics of cells in pleural effusion have identified some new biomarkers for molecular diagnosis [4–6]. In order to further verify the quality of new molecular diagnostic markers, a series of studies, such as the design and implementation of prospective, comparable studies and cost-effectiveness studies, are still needed before these molecular level findings can be applied to the clinical diagnosis of pleural effusion [7].

Hsp90 (heat shock protein 90) is thought to be a chaperone that helps some protein structures fold correctly, assists in protein resistance to heat stress, and plays a role in protein degradation. Because it helps stabilize many proteins involved in tumor growth, Hsp90 inhibitors are often used as anticancer drugs [8, 9]. There are five subtypes of Hsp90 that have been identified, and Hsp90-alpha and Hsp90-beta are the two major subtypes [5]. The homology of human Hsp90-alpha and Hsp90-beta is as high as 85% or more [10]. Previously, we found that the expression of Hsp90-beta was higher in lung cancer NCI-A549 and NCI-H446 cells than in human bronchial epithelial cells (16-HBE) and increased Hsp90-beta correlated with postoperative survival time and lymph node metastasis of lung cancer patients [11]. However, the expression of Hsp90-beta in the MPE of lung cancer patients is not clear. In this study, we tested the expression of Hsp90-beta in the MPE of lung cancer patients and compared with benign pleural effusion in order to assess the clinical significance of Hsp90-beta as a diagnostic marker for differential diagnosis of pleural effusion caused by lung cancer.

## Material and methods

### Ethics statement

This study was a non-blind, non-randomized and retrospective study. The process of research was conducted under the guideline of medical ethics principles, and all patients signed informed consent before being included in the study. The study was approved by Research Ethics Committees of research institutes (First Affiliated Hospital, Xi’an Medical University, Xi’an, China; Shenmu Hospital, Shenmu, China; Jining NO.1 People’s Hospital, Jining, China; Gansu Provincial Hospital, Lanzhou, China).

### Statement for strengthening the reporting of observational studies in epidemiology (STROBE)

Readers should be able to easily identify the intent and purpose of the study through the title and abstract of the study. The medical terms and keywords used in this report also help ensure proper indexing of articles in electronic databases. This study clearly describes the sample size, the variables examined and the observations. The intention of treatment analysis and outcome reporting bias analysis was also performed.

### Inclusion process for patients

From June 2015 to January 2018, a total 107 lung cancer patients with MPE were recruited into the investigation (First Affiliated Hospital, Xi’an Medical University, Xi’an, China; Shenmu Hospital, Shenmu, China; Jining NO.1 People’s Hospital, Jining, China; Gansu Provincial Hospital, Lanzhou, China). Before radiotherapy and chemotherapy, the samples of the patients were collected. After that, the patients were underwent thoracic catheter drainage and cisplatin was injected thoracic cavity for thoracic infusion chemotherapy (once a week). After at least 2 times of thoracic infusion, blood and pleural fluid samples were taken again. From June 2015 to January 2018, the pleural fluid and serum of 50 patients with benign pleural effusion (non-cancerous and non-tuberculous) were collected as a control. Clinical characteristics were retrieved from the clinical records available and were assessed retrospectively (Table 1).

**Table 1:** Clinico-pathological features of lung patients with MPE (*N* = 107)

Group	Characteristics	Lung cancer	Non-lung cancer
		Number (%)	Number (%)
Sex	Male	60 (56.1%)	30 (60%)
	Female	47 (43.9%)	20 (40%)
Age	≤ 60	38 (35.5%)	19 (38%)
	≥60	69 (64.5%)	31 (62%)
Smoking	Yes	35 (32.7%)	15 (30%)
	No	72 (67.3%)	35 (70%)
Histology	LAC	61 (57%)	
	LSCC	37 (34.6%)	
	SCLC	9 (8.4%)	
Pathologic grade	Poorly differentiated	73 (68.2%)	
	Moderately differentiated	15 (14%)	
	Well-differentiated	10 (9.4%)	
	Undifferentiated	9 (8.4%)	
T stage	T2	15 (14%)	
	T3	20 (18.7%)	
	T4	63 (58.9%)	
	Unavailable	9 (8.4%)	
Lymphatic invasion	N1	10 (9.3%)	
	N2	25 (23.4%)	
	N3	63 (58.9%)	
	Unavailable	9 (8.4%)	

*LAC* lung adenocarcinoma, *LSCC* lung squamous cell carcinoma, *SCLC* small cell lung cancer, *T stage* size of tumor

### Diagnostic criteria of MPE

MPE caused by lung cancer must diagnosed with the following two criteria [12, 13]: (1) Primary bronchogenic lung cancer must be confirmed by pathology through bronchoscopic biopsy, percutaneous lung biopsy and surgical resection biopsy and (2) MPE must be pathologically confirmed by thoracoscopic pleural biopsy and pleural fluid cytopathology.

### Inclusion criteria of MPE patients

Inclusion criterion: (1) The diagnosis of MPE must meet the diagnostic criteria of MPE; (2) No thoracic infusion chemotherapy or systemic chemotherapy was performed within 30 days before the initial collection of the specimens; (3) The routine blood test of the included patient was normal, and there was no obvious abnormal change of liver and kidney function; (4) No fever and other chronic medical diseases; (5) The Kamofsky (KPS) score of the patient was greater than or equal to 60 points and (6) The patient signed the informed consent.

### Exclusion criteria of MPE patients

Exclusion criteria: (1) Pleural effusion caused by tuberculous pleurisy; (2) Combined with interstitial lung disease, empyema, pneumonia, bronchial pneumonia and other pulmonary organic disease; (3) Combined with diabetes, mental illness, and cognitive dysfunction obstacles, and (4) hematopathy and other autoimmune diseases.

### Blood sample collection

All patients and control individuals were taken fasting 5 mL venous blood and placed in heparin anticoagulant tube. After half an hour at room temperature, the blood was centrifuged at 1000 rpm for 5 min. The supernatant was immediately loaded in Eppendorf tube and stored in a refrigerator at − 70 °C. The frozen serum was defrosted at room temperature when testing.

### Pleural effusion catheter drainage

We used the ultrasound-guided two-step method (Seldinger) to complete the thoracic catheterization. The specific steps are as follows: (1) The operator pierced the puncture needle into the pleural effusion through ultrasonic guidance, then pulled out the needle core and drew a small amount of fluid; (2) After that, the operator inserted the guide wire, then pulled the needle sheath, and expanded the needle channel with the expansion pipe; subsequently, the drainage tube was inserted into the thoracic cavity along the guide wire; and (3) The drain bag was attached to the drain pipe and the drain pipe was fixed properly.

### Treatment of pleural effusion specimen

A pleural effusion of approximately 20 mL was drawn through the thoracic drainage tube and indwelled with an ethylene diamine tetraacetic acid (EDTA) anticoagulant tube. The indwelling pleural fluid was centrifuged at 3000 rpm for 20 min. The supernatant was stored in 2 mL Eppendorf tube and stored in a low-temperature refrigerator at − 70 °C for testing.

### Cisplatin thoracic perfusion chemotherapy

After draining the pleural effusion as much as possible, we infused physiological saline 40 mL, cisplatin 40 mg/m^2^, and dexamethasone 5 mg sequentially through the patient’s chest drainage tube. Heparin sealed and fixed after injection. The frequency of thoracic infusion chemotherapy is once a week, and at least 2 times perfusion treatment.

### Enzyme-linked immunosorbent assay (ELISA)

The levels of Hsp90-beta in serum and MPE were detected by a method of sandwich ELISA, which was originally developed using rabbit anti-human Hsp90-beta antibody. The test was performed according to the manufacturer’s instructions (Xitang Biotech, Shanghai, China) [10, 14]. First, the 96-hole flexible micro titration plate (xitang biotechnology co., LTD., Shanghai, China) was coated with a serum (or MPE) sample diluted by PBS (pH 7.4) containing 1% BSA. After that, the hole was incubated with the monoclonal anti-Hsp90-beta antibody (10 ng/mL) at 37 °C for 1 h, then followed by reaction with avidin-conjugated peroxidase (Dako Cytomation) using a Substrate Reagent (R&D Systems) at 37 °C for 15 min. Finally, the chromogenic reaction was terminated by adding 50 μL 2 N sulfuric acid, and the intensity was measured at 450 nm wavelength by photometer. The standard curve of each plate was drawn using the concentration of the standard sample and the corresponding OD value of each hole.

### Statistical analysis

We used the SPSS 23.0 software package (SPSS Institute, version 21.0, Chicago, USA) to perform relevant statistical analysis. The comparison for levels of Hsp90-beta in serum and MPE were analyzed using the Student’s *T*-test, One–WAY ANOVA and Kruskal Wallis Test. The statistical results of the measurement data were expressed as mean ± standard deviation (X ± S). We constructed a receiver operating characteristic (ROC) curve to determine the specificity and sensitivity of Hsp90-beta in MPE for identifying lung cancer. All statistics were conducted using a bilateral test and statistical significance was set at *P* < 0.05.

## Results

### MPE of lung cancer patients shows an increased Hsp90-beta level compared with pleural effusion of control individuals

**Fig. 1:**
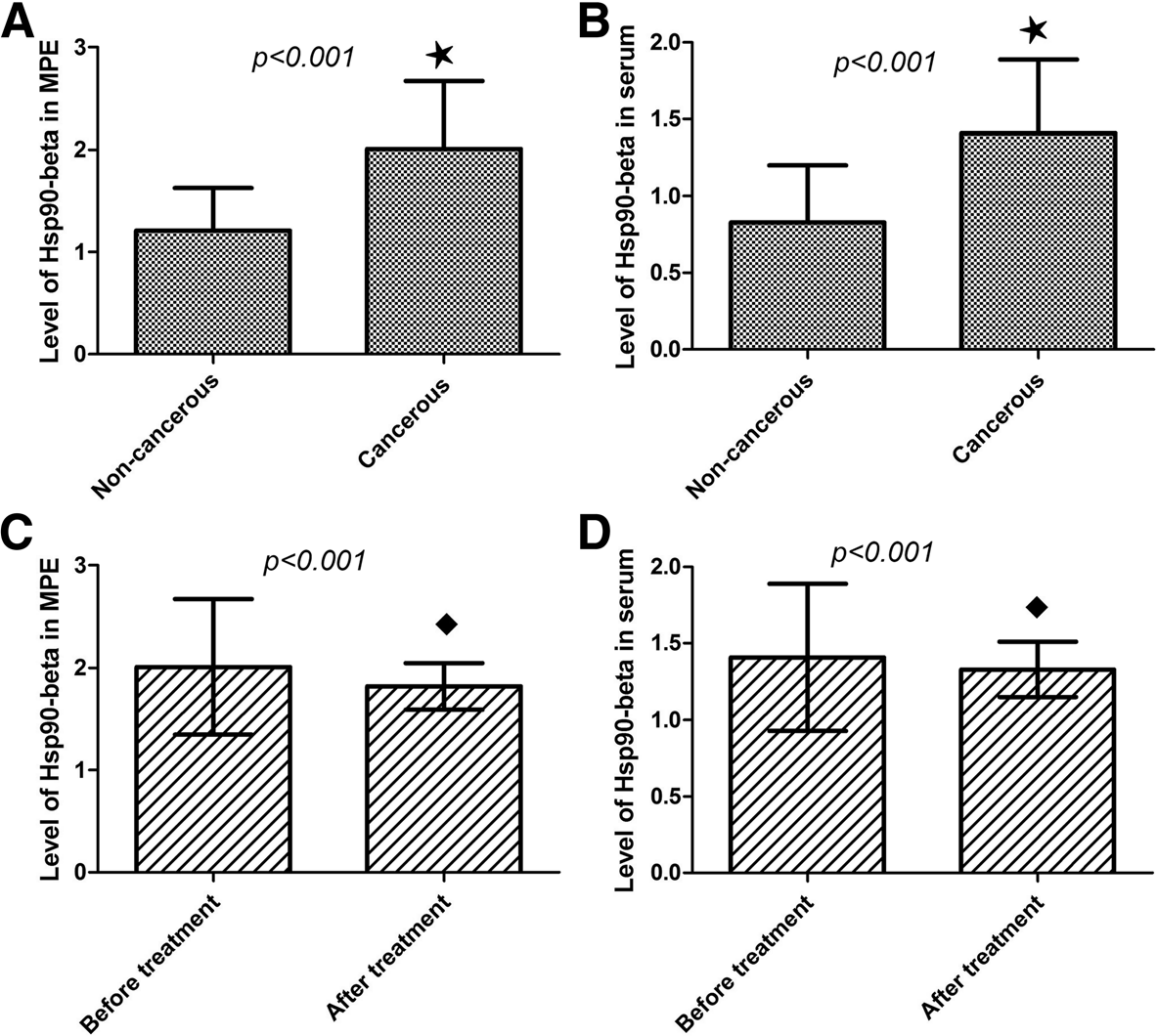
Expression levels of Hsp90-beta in MPE and serum of lung cancer patients. **a** High level of Hsp90-beta was observed in MPE of lung cancer patients compared with in MPE of control individuals (^★^, *P* < 0.001). **b** High expression of Hsp90-beta was showed in serum of lung cancer patients compared with in serum of control individuals (^★^, *P* < 0.001). **c** Thoracic perfusion of cisplatin significantly down-regulated the Hsp90-beta expression in MPE of patients with lung cancer (^■^, *P* < 0.001). **d** Thoracic perfusion of cisplatin significantly down-regulated the Hsp90-beta expression in serum of patients with lung cancer (^■^, *P* < 0.001). MPE, malignant pleural effusion

As shown in Table 2, increased level of Hsp90-beta was observed in MPE of the 107 lung cancer patients (2.01 ± 0.66 ng/mL) compared with pleural effusion of the 50 control individuals (1.21 ± 0.42 ng/mL) (*P* < 0.001) (Fig. 1a). In addition, increased level of Hsp90-beta was observed in serum of the lung cancer patients (1.41 ± 0.48 ng/mL) compared with in serum of the control individuals (0.83 ± 0.37 ng/mL) (*P* < 0.001) (Fig. 1b).

**Table 2:** Expression level of Hsp90-beta in MPE and serum in lung cancer patients with MPE and correlation with cisplatin thoracic perfusion therapy

	Items	*N*	Level of Hsp90-beta in MPE				Level of Hsp90-beta in serum			
			M ± SD(ng/ml)	DF	Statistical value	*P*	M ± SD(ng/ml)	DF	Statistical value	*P*
Resource	Non-cancerous	50	1.21 ± 0.42	155	*T* = −18.46	< 0.001	0.83 ± 0.37	155	*T* = −13.39	< 0.001
	Cancerous	107	2.01 ± 0.66^a^				1.41 ± 0.48^a^			
Groups	Before treatment	10	2.01 ± 0.66	2	F = 10.591	< 0.001	1.41 ± 0.48	2	F = 11.302	< 0.001
(Cisplatin perfusion)	After treatment	10	1.82 ± 0.23^b^				1.33 ± 0.18^b^			

*MPE* malignant pleural effusion; ^a^: cancerous compared with benign and non-cancerous; ^b^: after treatment compared with before treatment; *M ± SD* mean ± standard deviation, *DF* degree of freedom

### Cisplatin infusion therapy down-regulates the level of Hsp90-beta in MPE of lung cancer patients

As shown in Table 2, thoracic perfusion of cisplatin down-regulated the Hsp90-beta level in MPE of lung cancer patients (2.01 ± 0.66 ng/mL versus 1.82 ± 0.23 ng/mL) (*P* < 0.001) (Fig. 1c). Moreover, compared to before treatment (1.41 ± 0.48 ng/mL), Hsp90-beta level in serum of patients with lung cancer also showed a decrease (1.33 ± 0.18 ng/mL) (*P* < 0.001) (Fig. 1d), but the decline was much less than that in MPE.

### Increased Hsp90-beta in MPE of lung cancer patients correlates with pathologic grade of lung cancer

**Fig. 2:**
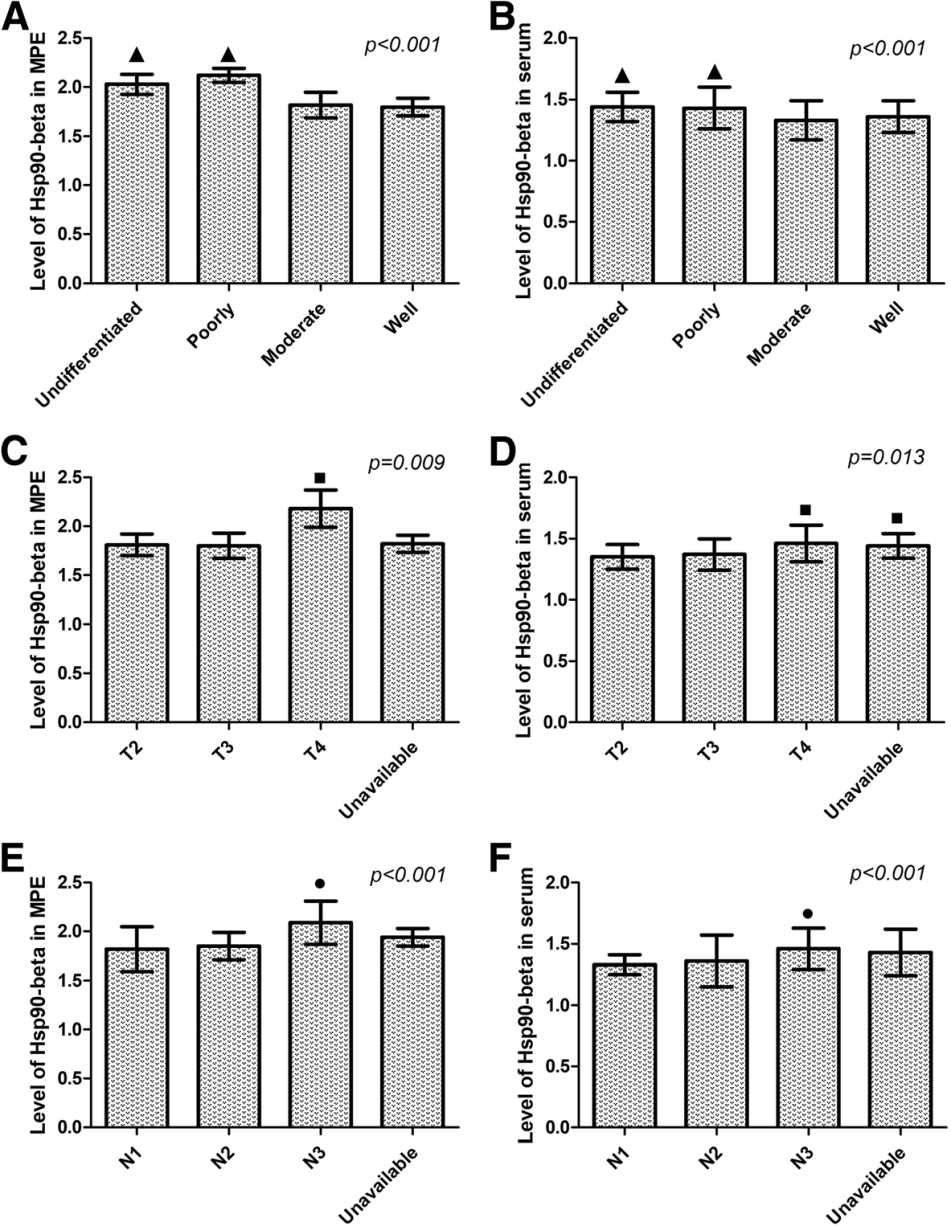
Correlation between clinicopathologic factors of lung cancer patients and the expression levels of Hsp90-beta in MPE and serum. **a**, **b** Poorly and undifferentiated patients revealed an up-regulated level of Hsp90-beta in MPE and serum than moderate and well differentiation (^▲^, *P* < 0.001); **c**, **d** The levels of Hsp90-beta in MPE and serum of lung cancer patients at T4 stage was significantly higher than that in patients at T3 stage and T2 (^■^, *P* = 0.009, =0.013). **e**, **f** Lymphatic invasion patients at N3 stage revealed an increased level of Hsp90-beta in MPE and serum than in those at N2 and N1 stage. MPE, malignant pleural effusion; T, the size of tumor; N, classification of lymph node metastases

As shown in Table 3, whether in MPE or serum, patients with undifferentiated (2.03 ± 0.10 ng/mL in MPE; 1.44 ± 0.12 ng/mL in serum) and poorly undifferentiated (2.12 ± 0.07 ng/mL in MPE; 1.43 ± 0.17 ng/mL in serum) lung cancer showed an increased Hsp90-beta level compared with those of moderate (1.82 ± 0.13 ng/mL in MPE; 1.33 ± 0.16 ng/mL in serum) and well differentiation (1.80 ± 0.09 ng/mL in MPE; 1.36 ± 0.13 ng/mL in serum) (*P* < 0.001) (Fig. 2a and b).

**Table 3:** Correlation between the level of Hsp90-beta in MPE and serum in lung cancer patients with MPE and clinicopathologic factors

Groups	Items	N	Level of Hsp90-beta in MPE				Level of Hsp90-beta in serum			
			M ± SD(ng/mL)	DF	Statistical value	*P*	M ± SD(ng/mL)	DF	Statistical value	*P*
Sex	Male	60	1.98 ± 0.36	105	*T* = −0.78	0.63	1.39 ± 0.16	105	*T* = −0.82	0.09
	Female	47	2.13 ± 0.49				1.42 ± 0.09			
Age	< 60	38	1.92 ± 0.16	105	*T* = −1.48	0.14	1.38 ± 0.18	105	*T* = −1.49	0.12
	≥60	69	2.01 ± 0.19				1.42 ± 0.13			
Smoking	Yes	35	1.99 ± 0.26	105	*T* = −0.878	0.38	1.40 ± 0.19	105	T = −0.878	0.382
	No	72	2.06 ± 0.31				1.41 ± 0.17			
Histology	LAC	61	2.00 ± 0.19	2	F = 1.503	0.227	1.41 ± 0.19	2	F = 1.602	0.231
	LSCC	37	1.98 ± 0.11				1.39 ± 0.14			
	SCLC	9	2.03 ± 019				1.44 ± 0.22			
Pathologic grade	Undifferentiated	9	2.03 ± 0.10^a^	3	F = 9.370	< 0.001	1.44 ± 0.12^a^	3	F = 9.370	< 0.001
	Poorly	73	2.12 ± 0.07^a^				1.43 ± 0.17^a^			
	Moderate	15	1.82 ± 0.13				1.33 ± 0.16			
	Well	10	1.80 ± 0.09				1.36 ± 0.13			
T stage	T2	15	1.81 ± 0.11	2	F = 9.996	0.009	1.35 ± 0.10	2	F = 3.778	0.013
	T3	20	1.80 ± 0.13				1.37 ± 0.13			
	T4	63	2.18 ± 0.19^b^				1.46 ± 0.15^b^			
	Unavailable	9	1.82 ± 0.09				1.44 ± 0.10^c^			
Lymphatic invasion	N1	10	1.82 ± 0.23	2	F = 10.591	< 0.001	1.33 ± 0.08	2	F = 11.302	< 0.001
	N2	24	1.85 ± 0.14				1.36 ± 0.21			
	N3	63	2.09 ± 0.22^c^				1.46 ± 0.17^c^			
	Unavailable	10	1.94 ± 0.09				1.43 ± 0.19			

MPE malignant pleural effusion, *LAC* lung adenocarcinoma, *LSCC* lung squamous cell carcinoma, *SCLC* small cell lung cancer; ^a^: undifferentiated and poorly compared with moderate and well; ^b^: T4 compared with T3 and  T2; ^c^: N3 compared with N2 and N1; *M ± SD* mean ± standard deviation, *DF* degree of freedom

### Increased Hsp90-beta in MPE of lung cancer patients correlates with tumor size of lung cancer

As shown in Table 3, the level of Hsp90-beta in MPE and serum of lung cancer patients at T4 stage (2.18 ± 0.19 ng/mL in MPE; 1.46 ± 0.15 ng/mL in serum) was significantly higher than that in patients at T3 stage (1.80 ± 0.13 ng/mL in MPE; 1.37 ± 0.13 ng/mL in serum) and T2 (1.81 ± 0.11 ng/mL in MPE; 1.35 ± 0.10 ng/mL in serum) (*P* = 0.009, = 0.013) (Fig. 2c and d).

### Increased Hsp90-beta in MPE of lung cancer patients correlates with lymphatic invasion of lung cancer patients

As shown in Table 3, the level of Hsp90-beta in MPE and serum of lung cancer patients with lymphatic invasion of N3 (2.09 ± 0.22 ng/mL in MPE; 1.46 ± 0.17 ng/mL in serum) was significantly higher than that in patients with N2 (1.85 ± 0.14 ng/mL in MPE; 1.36 ± 0.21 ng/mL in serum) and N1 (1.82 ± 0.23 ng/mL in MPE; 1.33 ± 0.08 ng/mL in serum) (*P* < 0.001) (Fig. 2e and f).

### Hsp90-beta level shows a strong positive correlation between in MPE and serum of patients with lung cancer

**Fig. 3:**
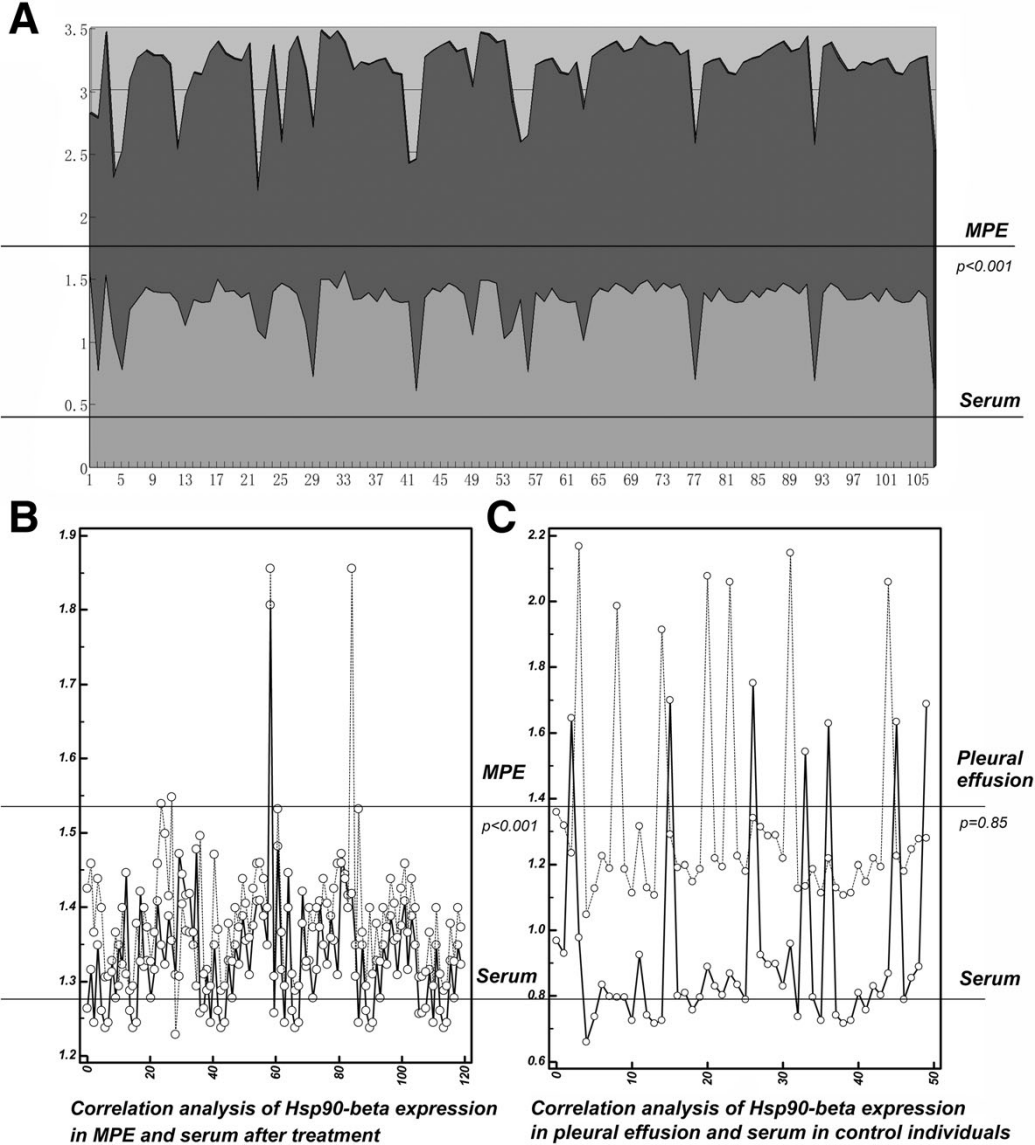
Correlation between MPE level of Hsp90-beta and serum level of lung cancer patients. **a** A clear positive correlation was observed between the expression of Hsp90-beta in serum and MPE in patients with lung cancer before treatment (*P* < 0.001). **b** After pleural infusion therapy of cisplatin, the levels of Hsp90-beta in serum and MPE in patients with lung cancer did not have a clear correlation (*P* = 0.85). **c** A positive correlation was also showed between the expression of Hsp90-beta in serum and pleural effusion in control individuals (*P* < 0.001)

There was a clear positive correlation between the levels of Hsp90-beta in serum and MPE in patients with lung cancer before thoracic infusion chemotherapy (Pearson correlation = 1.000; *P* < 0.001) (Fig. 3a). However, after pleural infusion therapy of cisplatin, the level of Hsp90-beta in serum and MPE did not show a correlation (Pearson correlation = 0.028; *P* = 0.85) (Fig. 3b). In control individuals, the level of Hsp90-beta in serum and MPE also showed a positive correlation (Pearson correlation = 1.000; *P* < 0.001) (Fig. 3c).

### Cut-off value of MPE Hsp90-beta for differentiating lung cancer patients from control individuals

The cut-off value for MPE Hsp90-beta was determined based on ROC curve analysis [15]. The maximum value of Hsp90-beta was 2.376 ng/mL and located in the MPE. The minimum was 0.608 ng/mL and distributed in the serum of control group. As shown in Table 4, the cut-off value of serum Hsp90-beta for differentiating lung cancer patients from control individuals located in the segment of 1.2–1.4 ng/mL and 95% confidence interval (CI) were 3.254 to 161.134; the cut-off value of MPE Hsp90-beta located in the segment of 1.6–1.8 ng/mL and 95% CI were 3.19 to 36.98.

**Table 4:** NLR and PLR of Hsp90-beta serum level in lung cancer patients and control individuals

Interval	Positive	Negative	Likelihood ratio	95% CI
NLR and PLR of serum Hsp90-beta for differentiating lung cancer patients from control individuals				
0.6–0.8	8	21	0.178	0.0848 to 0.374
0.8–1.0	0	16	0.000	0.000 to 0.239
1.0–1.2	9	5	0.841	0.297 to 2.381
1.2–1.4	49	1	22.897	3.254 to 161.134
1.4–1.6	41	1	19.159	2.712 to 135.355
1.6–1.8	0	6	0.000	0.000 to 0.684
Total	107	50		
NLR and PLR of MPE Hsp90-beta for differentiating lung cancer patients from control individuals				
1.0–1.2	3	20	0.0584	0.0185 to 0.185
1.2–1.4	4	17	0.0984	0.0353 to 0.274
1.4–1.6	8	2	17.33	6.4 to 26.78
1.6–1.8	15	4	25.87	3.19 to 36.98
1.8–2.0	71	2	21.963	5.638 to 85.550
2.0–2.2	4	5	0.374	0.105 to 1.333
2.2–2.4	1	0	∞	0.0319 to ∞
Total	107	50		

*NLR* negative likelihood ratio, *PLR* positive likelihood ratio, *95% CI* 95% confidence interval

### Accuracy of Hsp90-beta for predicting lung cancer

**Fig. 4:**
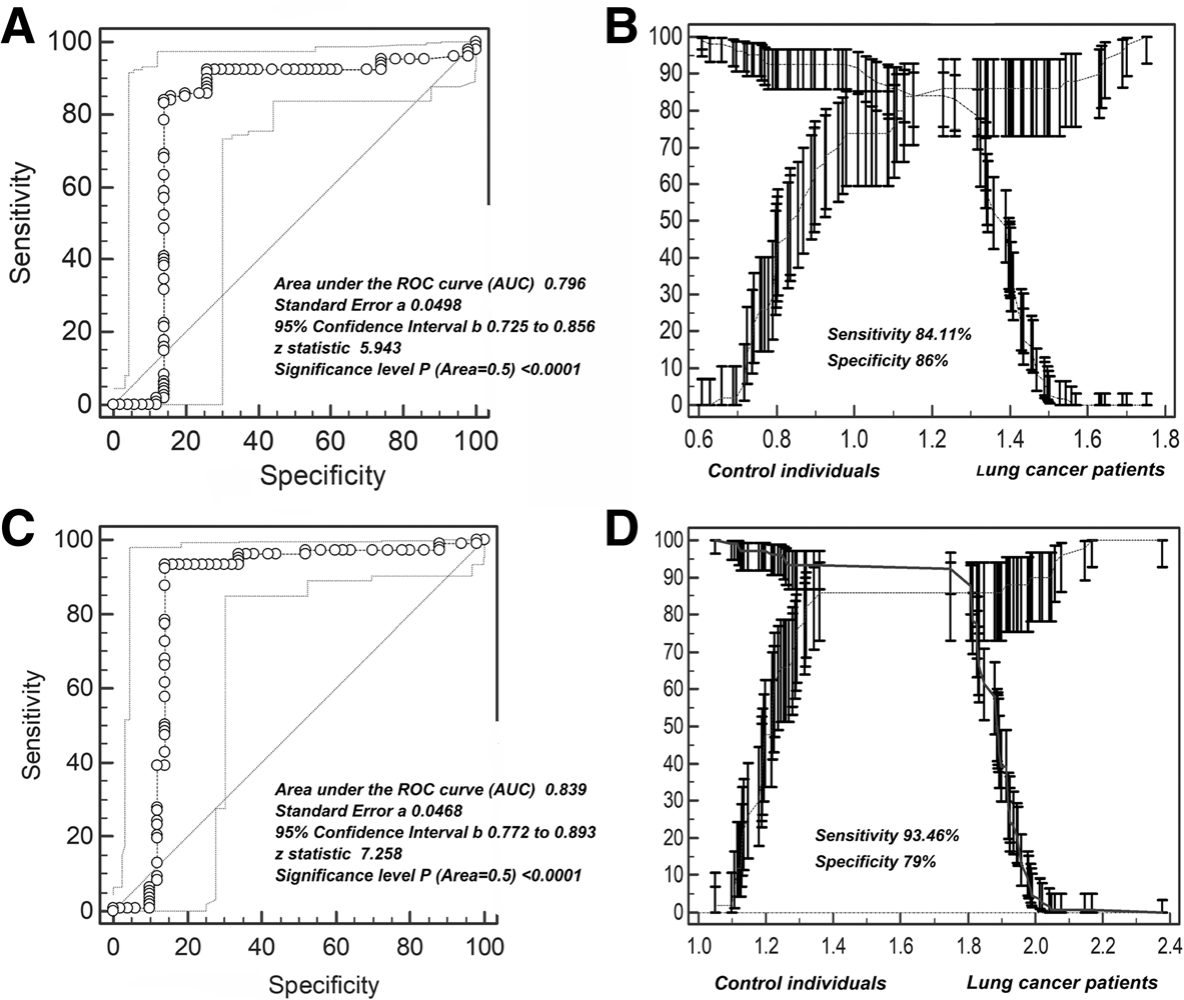
Cut-off selection of MPE Hsp90-beta by ROC curve analysis. **a**, **b** Selection of cut-off for serum value of Hsp90-beta in distinguishing lung cancer patients from control individuals (sensitivity 84.11%; specificity 86%); receiver operating characteristic curves for Hsp90-beta [area under the curve (auc): 0.796]. **c**, **d** Selection of cut-off for serum value of Hsp90-beta in distinguishing lung cancer patients from control individuals (sensitivity 93.46%; specificity 79%); receiver operating characteristic curves for Hsp90-beta [area under the curve (auc): 0.839]. ROC, receiver operating characteristic curve

As shown in Table 5, when compared lung cancer patients with control individuals using serum Hsp90-beta, the threshold was 1.228 ng/mL and the sensitivity and specificity were 84.11 and 86% respectively; when using MPE Hsp90-beta, the cut-off value of 1.659 ng/mL appealed a sensitivity of 93.46% and a specificity of 79%. Figure 4 (a and b) depicts ROC curve for serum Hsp90-beta, which shows that the area under the curve (AUC) was 0.796, standard error was 0.0498 (95% confidence interval: 0.725–0.856, Z value was 5.943 (*P* < 0.0001). Figure 4 (c and d) depicts ROC curve for MPE Hsp90-beta and displays an AUC of 0.839 with a standard error of 0.0468 (Z value = 7.258; *P* < 0.0001).

**Table 5:** Cut-off score of Hsp90-beta level for differentiating lung cancer patients from control individuals

Criterion	Sensitivity	95% CI	Specificity	95% CI
Cut-off score for serum Hsp90-beta for differentiating lung cancer patient from control individuals				
> =0.608	100.00	96.6–100.0	0.00	0.0–7.1
> 0.888	92.52	85.8–96.7	60.00	45.2–73.6
> 0.978	92.52	85.8–96.7	74.00	59.7–85.4
> 1.228 *	84.11	75.8–90.5	86.00	73.3–94.2
> 1.468	8.41	3.9–15.4	86.00	73.3–94.2
> 1.689	0.00	0.0–3.4	96.00	86.3–99.5
> 1.752	0.00	0.0–3.4	100.00	92.9–100.0
Cut-off score for MPE Hsp90-beta for differentiating lung cancer patient from control individuals				
> =1.048	100.00	96.6–100.0	0.00	0.0–7.1
> 1.188	97.20	92.0–99.4	38.00	24.7–52.8
> 1.286	93.46	87.0–97.3	71.00	57.5–83.8
> 1.659 *	93.46	87.0–97.3	79.00	71.3–90.2
> 1.786	50.47	40.6–60.3	86.00	73.3–94.2
> 1.988	5.61	2.1–11.8	90.00	78.2–96.7
> 2.058	0.93	0.02–5.1	94.00	83.5–98.7

*95% CI* 95% confidence; *cut-off value

## Discussion

Lung cancer often invades the pleura, resulting in the metastasis of cancer cells in pleura, in most cases can form malignant pleural effusion (MPE) [4]. It is generally believed that MPE is mainly caused by the dysfunction of the pleural wall or the poor drainage of lymph fluid. However, lymphatic obstruction does not fully explain MPE, and pleural inflammation and vascular leakage are currently considered to be also involved [16]. Recently, the diagnostic accuracy of MPEs has been improved by the development of new chest imaging modalities [17]. However, in most cases it is necessary to confirm the presence of malignant cells in the pleural and pleural fluids to determine the diagnosis of MPE. Some conventional tumor markers in MPE such as carcinoembryonic antigen (CEA), carbohydrate antigen (CA) -153 and cytokeratin 19 fragment (CYFRA 21-1) appear to show a certain diagnostic value [4, 16]. Previously, we found that Hsp90-beta was higher in H446 cells (1.72 times) and A549 cells (2.19 times) than 16-HBE cells [18] and that increase of Hsp90-beta correlated with postoperative survival time and lymph node metastasis of lung cancer patients [11]. In this study, we measured the level of Hsp90-beta in MPE of lung cancer patients and assessed the clinical value of Hsp90-beta as MPE diagnostic indicator.

We found that whether in MPE or serum of lung cancer patients, the level of Hsp90-beta was higher than in that of control individuals. In addition, the elevation of Hsp90-beta in MPE was greater than that in serum, which indicated that the high level of hsp90-beta in MPE may reflect the occurrence and progress of lung cancer to some extent. For example, high level of Hsp90-beta has shown to be up-regulated in several cancers, such as oropharyngeal squamous cell carcinoma, breast and lung carcinomas [19–22]. Previous study reports that the expression of Hsp90-beta in gastric cancer tissues was higher than that in non-cancerous gastric mucosa, especially in poorly differentiated cancer tissues [23]. In our study, we especially noticed that thoracic perfusion of cisplatin down-regulated the Hsp90-beta level in MPE of patients with lung cancer. Cisplatin is used to treat many human cancers, including bladder, head and neck, lung, ovarian and testicular. Its anti-cancer mechanism is related to its ability to cross-link with purines in the DNA of cancer cells, which interferes with DNA repair mechanisms, causing DNA damage and inducing apoptosis of cancer cells [24]. One previous study indicates that cisplatin has a high affinity for Hsp90 and the binding of cisplatin to Hsp90 causes a conformational change in the protein. Although Hsp90 inhibits the aggregation of citrate synthase as a molecular chaperone in vitro, its activity is almost completely inhibited in the presence of cisplatin [25]. The chaperone activity of Hsp90 is affected by Mg/ATP. The cisplatin binding region of Hsp90 is close to the c terminal, and the combination of the two may inhibit the chaperone activity of Hsp90 through some complex mechanisms [25]. Although the selectivity of cisplatin in killing rapidly proliferating cancer cells depends largely on the covalent binding of cisplatin’s chloride site to DNA. One study suggests that cisplatin may also be toxic in cells with slow proliferation or terminal differentiation through drug-protein interactions and finds several proteins that bind to cisplatin, including glucose regulatory protein 94 (GRP94) and Hsp90 [26]. Future studies on the interaction between Hsp90 and cisplatin will be helpful for disclosing tumor sensitivity or resistance to cisplatin therapy.

In addition, we found that the level of Hsp90-beta in MPE of poorly differentiated lung cancer patients was higher than that in moderate and well differentiated lung cancer patients. The differentiation degree of lung cancer cells is an important reference data in lung cancer diagnosis and treatment. The worse the differentiation of cancer cells, the higher the degree of malignancy, the faster the tumor growth, and the more likely the lung cancer is to metastasize [3, 11, 18]. Our results indicate that the up-regulation of Hsp90-beta in MPE may be a molecular marker for development of MPE. Besides, we also found that over-expression of Hsp90-beta in MPE seems to be closely correlated with the tumor size and lymphatic invasion of lung cancer. Tumors with a higher degree of malignancy often develop faster and the tumor volume increases more rapidly and often accompanied by lymph node invasion and metastasis [18]. Our findings suggest that Hsp90-beta may play an important role in progress of lung cancer. Many studies have shown that the expression level of Hsp90-beta is closely related to the prognosis of cancer patients, indicating that over-expression of Hsp90-beta promotes the progression of malignant tumors and may be a prognostic indicator of malignant tumors [11, 14, 27–29].

Our investigation showed over-expression of MPE Hsp90-beta significantly correlated with malignant biological behavior of lung cancer of lung cancer patients, indicating that the high level of Hsp90-beta is not only closely related to the development of pleural effusion, but also a risk factor for the progression of lung cancer. A study claims that overall survival rate of lung cancer patients with high Hsp90-beta expression is lower than that of patients with low Hsp90-beta expression, suggesting that Hsp90-beta is an independent prognostic factor for lung cancer [30]. In addition, the over-expression of Hsp90-beta is also found in lung adenocarcinoma [29] and is closely related to the low differentiation of lung cancer, short overall survival and lymphatic infiltration. Another study **s**hows that Hsp90-beta is highly expressed in lung adenocarcinoma than in lung squamous cell carcinoma and is associated with low survival rate of patients [31]. In our study, we verified that the level of Hsp90-beta had a positive linear relationship between MPE and serum. The cumulative probability diagram showed that most of the detection points of the two methods were close to or located on the line, which means that there is a strong correlation between MPE and serum. However, the down-regulation of Hsp90-beta expression by intrapleural cisplatin perfusion indirectly reflects that it is an effective local treatment.

Using the ROC curve analysis, we obtained that the diagnostic threshold of Hsp90-beta in MPE was 1.659 ng/mL (1.228 ng/mL in serum). With this threshold, the sensitivity and specificity of MPE Hsp90-beta for distinguishing lung cancer from control individuals was 93.46 and 76%. We found that the sensitivity in MPE Hsp90-beta in identifying lung cancer was higher than that in serum (84.11%), but the specificity was lower than that in serum (86%), which means that the screening ability of MPE is better than that of serum. So, we can see that the detection of Hsp90-beta levels in MPE may be helpful in differential diagnosis of pleural effusion caused by lung cancer. However, this study also had some shortcomings. First, our study included only Chinese patients, which may result in geographical and ethnic bias. Second, the study did not address molecular-level mechanisms of Hsp90-beta and did not explore the mechanism by which cisplatin interacts with Hsp90-beta. Further experimental investigations involving a larger number of samples and molecular mechanism are required to reach a more definitive conclusion. MPE is the most common complication of lung cancer. Exploring the role of Hsp90-beta will help elucidate the mechanism of MPE development and help design targeted therapy based on Hsp90-beta.

## Conclusion

The level of Hsp90-beta was increased in MPE of lung cancer patients and up-regulation of Hsp90-beta was correlated with low differentiation, tumor size and lymphatic metastasis. In addition, the concentration of MPE Hsp90-beta presented a better sensitivity and moderate specificity in differential diagnosis of pleural effusion, suggesting that it has potential utility as a diagnostic, prognostic, and predictive tool for MPE. However, studies on larger numbers of subjects are required for higher evidentiary strength.

## Abbreviations

CA: Carbohydrate antigen
CEA: Carcinoembryonic antigen
CYFRA 21-1: Cytokeratin 19 fragment
DNA: Deoxyribonucleic acid
EDTA: Ethylene diamine tetraacetic acid
ELISA: Enzyme-linked immunosorbent assay
GRP94: Glucose regulatory protein 94
HBE: Human bronchial epithelial cells
Hsp90: Heat shock protein 90
KPS: Kamofsky score
LAC: Lung adenocarcinoma
LSCC: Lung squamous cell carcinoma
MDR: Multidrug resistance
MPE: Malignant pleural effusion
ROC: Receiver operating characteristic
SCLC: Small cell lung cancer
STROBE: Statement for strengthening the reporting of observational studies in epidemiology
